# Cost-Effectiveness of a Telephone-Delivered Intervention for Physical Activity and Diet

**DOI:** 10.1371/journal.pone.0007135

**Published:** 2009-09-25

**Authors:** Nicholas Graves, Adrian G. Barnett, Kate A. Halton, Jacob L. Veerman, Elisabeth Winkler, Neville Owen, Marina M. Reeves, Alison Marshall, Elizabeth Eakin

**Affiliations:** 1 Queensland University of Technology, Institute for Health & Biomedical Innovation, Brisbane, Queensland, Australia; 2 The University of Queensland, School of Population Health, Brisbane, Queensland, Australia; Universidad Peruana Cayetano Heredia, Peru

## Abstract

**Background:**

Given escalating rates of chronic disease, broad-reach and cost-effective interventions to increase physical activity and improve dietary intake are needed. The cost-effectiveness of a Telephone Counselling intervention to improve physical activity and diet, targeting adults with established chronic diseases in a low socio-economic area of a major Australian city was examined.

**Methodology/Principal Findings:**

A cost-effectiveness modelling study using data collected between February 2005 and November 2007 from a cluster-randomised trial that compared Telephone Counselling with a “Usual Care” (brief intervention) alternative. Economic outcomes were assessed using a state-transition Markov model, which predicted the progress of participants through five health states relating to physical activity and dietary improvement, for ten years after recruitment. The costs and health benefits of Telephone Counselling, Usual Care and an existing practice (Real Control) group were compared. Telephone Counselling compared to Usual Care was not cost-effective ($78,489 per quality adjusted life year gained). However, the Usual Care group did not represent existing practice and is not a useful comparator for decision making. Comparing Telephone Counselling outcomes to existing practice (Real Control), the intervention was found to be cost-effective ($29,375 per quality adjusted life year gained). Usual Care (brief intervention) compared to existing practice (Real Control) was also cost-effective ($12,153 per quality adjusted life year gained).

**Conclusions/Significance:**

This modelling study shows that a decision to adopt a Telephone Counselling program over existing practice (Real Control) is likely to be cost-effective. Choosing the ‘Usual Care’ brief intervention over existing practice (Real Control) shows a lower cost per quality adjusted life year, but the lack of supporting evidence for efficacy or sustainability is an important consideration for decision makers. The economics of behavioural approaches to improving health must be made explicit if decision makers are to be convinced that allocating resources toward such programs is worthwhile.

**Trial Registration:**

This paper uses data collected in a previous clinical trial registered at the Australian Clinical Trials Registry, Australian New Zealand Clinical Trials Registry: Anzcrt.org.au ACTRN012607000195459

## Introduction

Non-communicable diseases are projected to cause over three-quarters of deaths in 2030 [1]. Today more than 125 million Americans are living with chronic health conditions such as diabetes and cardiovascular disease and there are 1.7 million deaths annually [2]. High prevalence of these conditions are also seen in Australia and other industrialised countries [3]. Regular physical activity and a healthy diet are critical for the prevention and management of most chronic conditions [4].

In Australia, it has been estimated that for every 1% increase in the proportion of the population that becomes physically active, 100 deaths from coronary heart disease could be avoided, and $7.2 million in overall direct health care costs could be saved [5]. There is evidence for the effectiveness of interventions to improve physical activity and diet, in both primary and secondary prevention contexts [6]. This includes interventions delivered in community, health-care and workplace settings and via different intervention delivery modalities such as telephone, print and website [7]–[9].

Despite this evidence, a significant gap in the translation of research-into-practice remains, with only a small proportion of effective interventions adopted. Reasons for this might include a lack of dissemination research [10] or some mismatch between the information produced from randomised trials and the information required by decision-makers, who are constrained by scarce resources [11]. It has been argued that research is not being conducted to answer questions of importance to healthcare decision-makers; and, in particular, there is a dearth of high-quality economic analyses of health behaviour change intervention trials [12].

Reviews of cost-effectiveness studies on health behavior change interventions have identified significant gaps in knowledge. Dalziel et al. [13] identified five published cost-effectiveness studies and four cost-utility studies of physical activity interventions. While the authors of all studies reported that physical activity interventions were cost-effective, Dalziel suggested they were subject to methodological limitations. Gordon et al. [14] reviewed 64 studies on health behaviour interventions that address the major behavioural risk factors for chronic disease, including smoking, physical inactivity, poor diet and alcohol misuse. They found evidence of cost-effectiveness but highlighted heterogeneous study outcomes and methodological limitations. Müller-Riemenschneider et al. [15] considered 8 studies investigating 11 intervention strategies to promote physical activity behaviour in healthy adults. They found physical activity interventions were cost-effective but commented that appropriate cost-effectiveness analyses were rare and generalisability was limited.

There exists an evidence base for the efficacy of telephone-delivered interventions for physical activity and dietary behaviour change [7]. The aim of this paper is to describe a cost-effectiveness evaluation of a telephone-delivered intervention to improve physical activity and diet among adults with chronic conditions, recruited from Australian primary care practices. The methods [16] and behaviour-change outcomes of the intervention trial [17] have been reported and in this paper a decision analytic cost-effectiveness model is presented. The main research question addressed is: whether scarce healthcare resources should be invested toward a telephone counselling intervention that improves adults' dietary and physical activity behaviours, or, whether decision makers should remain with existing practice. Findings are interpreted with the information needs of health-care decision makers in mind.

## Materials and Methods

### Overview of the primary trial

A cluster-randomized trial of a telephone counselling intervention for physical activity and diet was conducted among adults from a low socio-economic community in Australia [17]. Data from 434 adult participants with type 2 diabetes or hypertension (mean age 58.2 [SD 11.8]; 61% female; mean BMI 31.1 [SD 6.8]) from a disadvantaged community in Queensland were used for cost-effectiveness modelling. Participants were recruited from ten primary care practices via electronic medical records searches for condition-eligible participants. Data were collected between February 2005 and November 2007 and analysed between January and October 2008.

Participants were randomised to Telephone Counselling (n = 228) or Usual Care (n = 206). Participants in the Telephone Counselling group received a 12-month intervention involving 18 telephone calls from trained counsellors. The intervention schedule was adapted from the work of King and colleagues to facilitate initiation and then maintenance of behaviour change [18], [19]. During the initiation phase, calls were delivered weekly for the first three weeks and then fortnightly up to four months. During the maintenance-enhancement phase, calls were made monthly between 4 and 12 months. Participants received an intervention workbook along with a pedometer, a self-monitoring form, and an exercise band. The workbook was adapted from the work of Demark-Wahnefried and colleagues [20]. To obtain approval from an ethics committee and to reduce attrition, the Usual Care group received more than would typically have been made available under existing practice conditions. Prior to the intervention, the Usual Care group were being managed by their general practitioner. They were consented into the trial and asked about their lifestyle behaviours over the course of the trial for the purpose of data collection. This involved three telephone interviews of approximately 45–60 minutes duration each. They were provided feedback on their behaviour after each assessment and sent off-the-shelf print materials about good health behaviour and a quarterly newsletter. The primary outcomes [17] show statistically-significant intervention effects (Telephone Counselling vs. Usual Care) from baseline to 12-months in: percentage calories from total fat (1.17% reduction); percentage energy from saturated fat (0.97% reduction); vegetable intake (increase 0.71 servings per week); fruit intake (increase 0.30 servings per week); and fiber (increase 2.23 grams per week). Increases in mean weekly physical activity at 12-months were reported by the Telephone Counselling (71 minutes) and Usual Care groups (84 minutes). Although to a lesser extent than the Telephone Counselling group, the Usual Care group experienced clinically minor but statistically significant improvements from baseline in intake of total fat, saturated fat and fruit.

### Intervention costs and valuing health outcomes

All costs are reported in 2008 Australian dollars. The cost of delivering the Telephone Counselling intervention and the Usual Care alternative were measured during the primary trial. Single-use items such as workbooks and mail-outs were attributed to participants. The jointly used resources of telephone counsellor time, fixed overheads and the staff costs of managing the program were allocated to participants on the basis of the number and duration of calls. All costs incurred were allocated across successful contacts. The dollar costs of input factors were obtained by a review of local market prices. The participant use of health care services between baseline and 12 months were self-reported and collected by validated questionnaire [21]. These included: consultations with a general practitioner, psychiatrist/psychologist; visits to an emergency department; visits by a nurse, home health nurse or occupational therapists; the costs of hospital admissions based on the number of bed days used; and, visits to an outpatient department. Dollar valuations of these resources were obtained from the Commonwealth Government schedule of re-imbursements [22]. The SF-36 health survey was administered to all participants at baseline, 4 months and 12 months. The data were mapped onto the SF-6D using a validated algorithm [23]. The SF-6D provides a preference-based value of health outcomes derived from standard gamble questions. The SF-6D is appropriate for estimating quality adjusted life years (QALY) [24].

### Health behaviours

Diet and physical activity outcomes of the trial were assessed using telephone interviews at baseline, 4-months and 12-months. Validated instruments used in Australian population health surveys were employed [25]–[27]. Diet and physical activity were assessed relative to Australian guidelines: 150 minutes a week of accumulated moderate physical activity on five or more days per week [28]; at least five servings per day of vegetables; at least two daily servings of fruit; less than 30% of energy intake from total fat; less than 10% of energy intake from saturated fat and 30 grams or more of fibre per day [29].

### Mortality risk

Risk of death was based on the estimated future mortality risks of the subjects in the trial. Self-reported data were collected on age, sex, body mass index, cigarette smoking status, history of diabetes mellitus or cardiovascular events, hypertension and hypercholesterolemia. The latter two were converted to systolic blood pressure and total serum cholesterol by imputing the age and sex-specific average values [30]. Data from the 2003 Australian Burden of Disease and Injury study [31] were used to describe the population in terms of disease-specific and overall mortality rates and these were adjusted according to each individual's risk factors. The adjusted mortality rates were used to estimate life expectancies of participants in a Sullivan life table [32].

### Cost-effectiveness model

**Figure 1:**
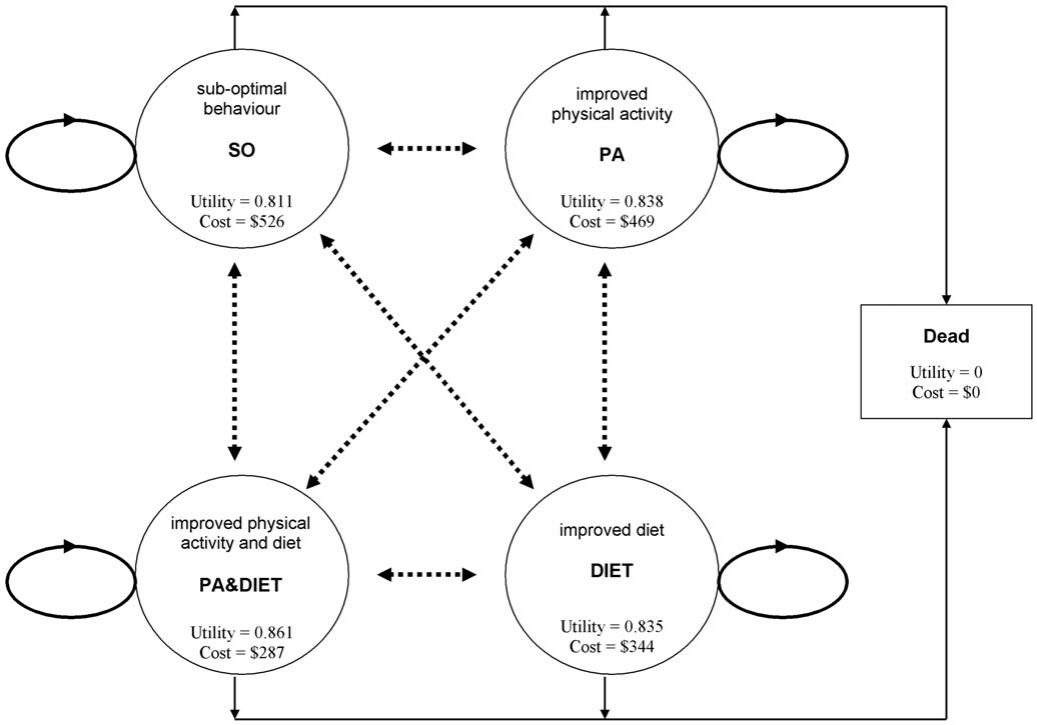
five-compartment state transition Markov model, with utilities and costs per person for the Telephone Counselling group in the first 12 months.

Cost-effectiveness was assessed using a state-transition Markov model, which predicted the progress of participants through five health states for ten years after recruitment. The structure of the model is shown in Figure 1. Individuals in a state of sub-optimal behaviour (SO) move to improved physical activity (PA) if they meet current Australian Physical Activity guidelines (more than 150 min/wk×5 days); or, move to improved diet (DIET) if they met at least three of the five current Australian recommended nutrition guidelines [29]. If both criteria are met then individuals move to improved physical activity and diet (PA&DIET). Participant movements through the health states are governed by the trial data on health behaviour and individual mortality risks. Risk of death increases with each cycle to reflect ageing and the modelling assumes – conservatively – that spending a cycle in a good health behaviour state does not reduce mortality risk. The model updates, and so individuals move, after one 4 month cycle and then at every 12 month time-point. Movements between all states are possible with the exception of the death state, which is absorbing [33]. The baseline to 4-month data informs the first transitions; the 4-month to 12-month data informs all subsequent transitions, until year 10. This introduces uncertainty, but allows costs and benefits arising in future time periods that were not observed within the timeframe of the trial, to be estimated. The observed cost and utility scores (SF-6D) of trial participants that occupied relevant health states for each model cycle were estimated from the trial data. They were then used to calculate the total cost and QALY outcomes for Telephone Counselling and Usual Care. Baseline values for costs and utilities from the whole sample were used to define an existing practice (Real Control) group. This group was assumed to make no changes to their health behaviours, costs or QALY outcomes over time. Costs and QALY outcomes accumulate as the model updates. To illustrate model output, we include the costs and utilities for the Telephone Counselling group at 12 months in Figure 1. The model is complete when all the costs and QALY outcomes for the intervention and comparators are simulated for a period of 10 years. All outcomes arising in future time periods are discounted at 3% [34]. A detailed description of the modelling method has been published [35].

### Model Evaluation

The first comparison, which is based on the cluster randomised trial, is Telephone Counselling vs. Usual Care. Comparison groups in randomised trials are often inappropriate comparators for cost-effectiveness analyses and decision making [36]. Usual Care did not equate to an existing practice alternative that is relevant to decision makers. To compensate for this a Real Control group was defined who represent a decision to remain with existing practice and can be thought of as ‘never contacted’ about their diet and exercise behaviour. The second comparison was Telephone Counselling vs. an existing practice alternative (Real Control). As the Usual Care group in the intervention received a ‘brief intervention’, the third comparison was Usual Care vs. existing practice alternative (Real Control). To simplify all comparisons, 100 participants were simulated in each group. The simulation was informed by all of the data reported in the primary trial (n = 431); three participants were excluded due to death.

The model shown in Figure 1 was fitted using the WBDiff interface of the WinBUGS software package (MRC Biostatistics Unit, Cambridge University and Imperial College, School of Medicine at St Mary's, London, 1989). Beta distributions were fitted for the parameters that described transition probabilities and health -related utilities, and gamma distributions were fitted for parameters that described costs [37]. The change in total cost (ΔC) and QALY outcomes (ΔE) were estimated for all three comparisons. These were combined with the decision maker's willingness to pay for QALYs (γ) to calculate changes to monetary net benefits:
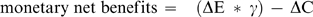



The probability an intervention was cost-effective vs. the relevant comparator was estimated by taking 2,000 random re-samples from the probability distributions for parameters; and then counting the number of times the monetary net benefit statistic was positive over the total number of re-samples [38]. The results of this process are plotted in the form of a cost-effectiveness acceptability curve that shows the probability a decision is cost-effective, given uncertainties in model parameters, for a range of the decision maker's willingness to pay for QALYs (γ). Fenwick et al. provides information about the use and interpretation of cost-effectiveness acceptability curves [38], [39].

## Results

**Figure 2:**
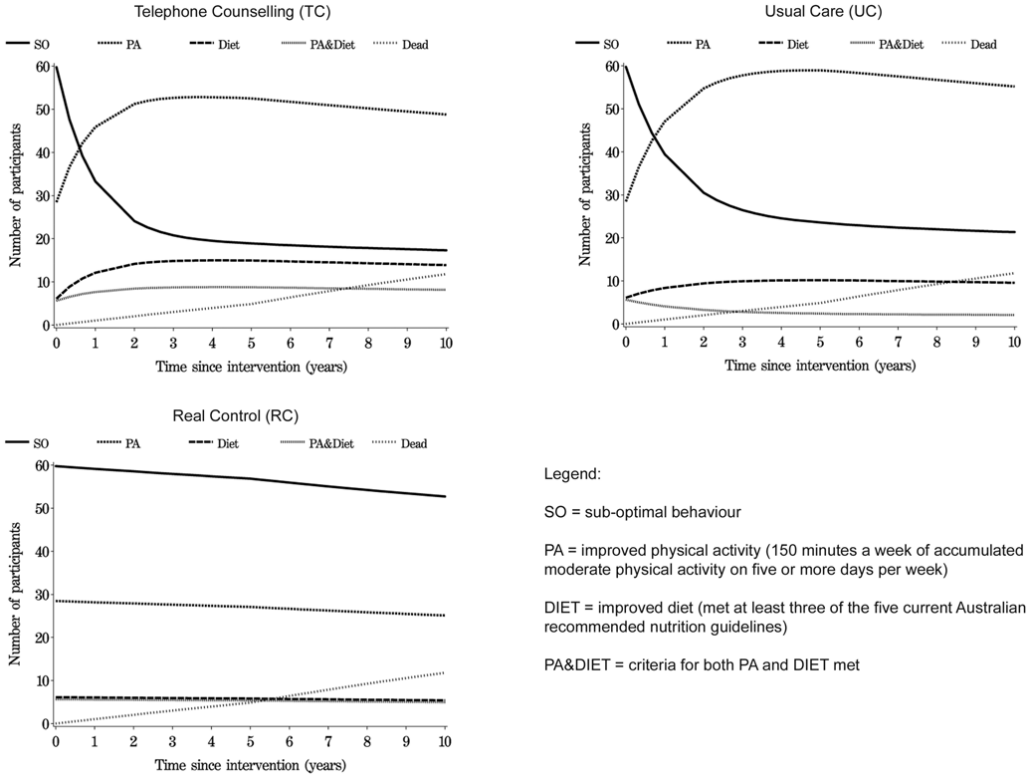
number of patients in different health states over time.

**Figure 3:**
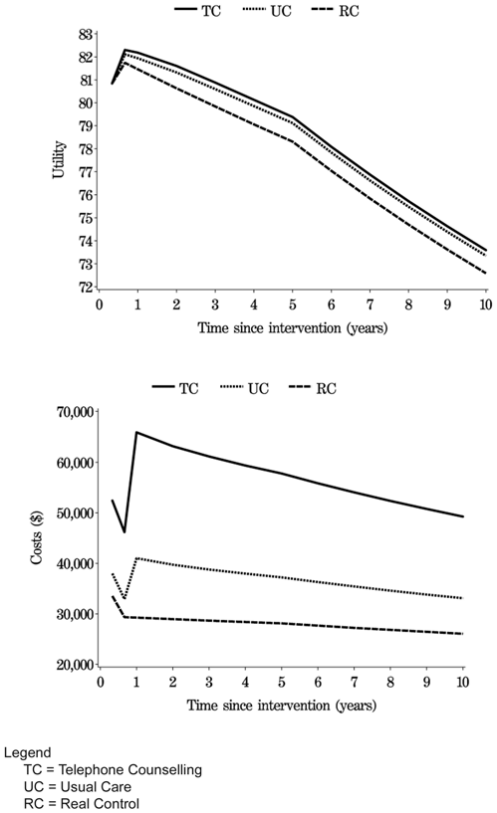
Total QALYs and costs over time.

The costs of delivering the Telephone Counselling intervention were estimated to be $570 for the first year and $410 per year for all subsequent years up to year 10. The costs of delivering Usual Care were estimated to be $134 per year for all 10 years. Plots that show membership of the Markov health states for Telephone Counselling, Usual Care and existing practice alternative (Real Control) over time are included in Figure 2. Because 100 participants were simulated in each group, the vertical sum of the five lines will always equal 100. Consistent with the increase in physical activity observed in the Usual Care group, the Telephone Counselling group spent only slightly more time in good health-behaviour states than did the Usual Care group, but substantially more time in good health behaviour states than did the existing practice alternative (Real Control) group. Risk of death was the same for all groups regardless of behaviour. Total QALYs and total costs, that include intervention costs and the cost of all accessing health care services, for all groups over time are shown in Figure 3.

The means and 95% Bayesian credible intervals of incremental cost (ΔC) and QALY (ΔE) outcomes for all comparisons are included in Table 1; a 95% Bayesian credible interval contains the true value with a 95% probability [40]. The results show that choosing the Telephone Counselling intervention over Usual Care costs $78,489 per QALY gained, choosing Telephone Counselling over the existing practice alternative (Real Control) costs $29,375 per QALY gained, and choosing Usual Care over existing practice alternative (Real Control) costs $12,153 per QALY gained. The results of the 2,000 re-samples that describe parameter uncertainty were transformed into monetary net benefits and used to plot cost-effectiveness acceptability curves.

**Table 1:** Means (95% Bayesian credible intervals) of re-sampled changes to cost and QALY outcomes; 100 individuals per group.

	Costs (Australian Dollars)	QALY
TC vs UC	$192,300 ($139,400, $217,800)	2.45 (–0.79, 5.97)
TC vs RC	$277,300 ($207,000, $302,000)	9.44 (6.42, 12.14)
UC vs RC	$84,950 ($62,030, $98,700)	6.99 (4.35, 9.55)

TC = Telephone Counselling.

UC = Usual Care.

RC = Real Control.

QALY = Quality Adjusted Life Year.

**Figure 4:**
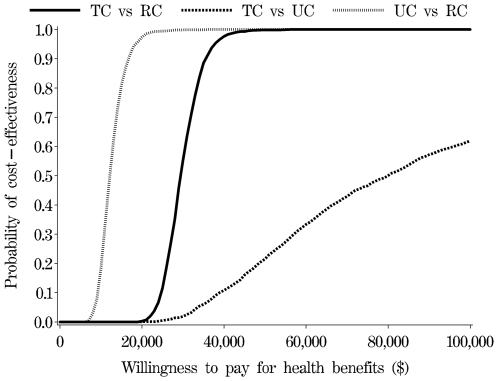
Cost-effectiveness acceptability curves.

Figure 4 shows the probability that the decisions evaluated are cost-effective (the y-axis), given joint uncertainty in model parameters, for different values of the decision maker's willingness to pay for health benefits (γ) (the x axis). The recommended value for decision making in the Australian setting is $64,000 per QALY [41]. At this value a decision to adopt Telephone Counselling over Usual Care has only a 38% probability of being cost-effective, while choosing Telephone Counselling as compared to remaining with current practice (Real Control) has a 100% probability of being cost-effective. Choosing Usual Care over current practice (Real Control) also has a 100% chance of being cost effective.

## Discussion

There are no published studies that describe the economics of changing health behaviour in the Australian setting and few published internationally which evaluate the use of telephone-delivered interventions, despite evidence for the effectiveness of this modality [7]. This analysis draws on data collected from a methodologically strong trial, uses an appropriate method for assessing cost-effectiveness, captures the role of uncertainty among model parameters, and presents the results in a format suitable for interpretation by decision makers.

The comparison arising from the cluster-randomised controlled trial is whether to adopt Telephone Counselling over Usual Care, but this choice is unlikely to be relevant to decision makers. The Usual Care ‘control’ group did not approximate existing practice, which decision makers might prefer to use as the comparator for alternate programmes. The Usual Care group underwent extensive health behaviour assessment as part of their trial participation, and to minimise attrition and comply with ethical standards, they received behavioural feedback and generic print materials. Improvements among the Usual Care participants may have arisen as a result of their participation in assessment [42] and receipt of a brief intervention. The improvements in health behaviours in the Usual Care group from this study were primarily driven by improvements in physical activity. For all dietary outcomes, the intervention effect at 12-months was significant (greater improvements in Telephone Counselling vs. Usual Care) and the Usual Care group, on average, failed to make clinically meaningful changes for any of the dietary outcomes [17]. A review of published studies of physical activity interventions conducted in the primary care setting found that eight out of 28 studies showed physical activity increases among control group participants, equivalent to at least 60 minutes of physical activity per week (Personal communication, Lauren Waters, July 28, 2009). In this review, the extent of the intervention provided to the control group was not associated with control group improvements. Instead it was higher frequency of assessment, use of interviewer-administered assessment, and inclusion of at-risk individuals (i.e. secondary prevention) that were thought to play a role. These three features are present in the cluster-randomised controlled trial used for this cost-effectiveness modelling. The Usual Care group improvements in the current trial may have been a function of the research context rather than the brief intervention.

A useful comparison for decision-making is between Telephone Counselling and existing practice (Real Control). The findings from this comparison show a decision to adopt Telephone Counselling over existing practice (Real Control) is cost-effective given current information. For full transparency, the brief intervention of Usual Care was compared with existing practice (Real Control); this revealed the lowest incremental cost-effectiveness ratio (Table 1) and highest probability of being the best decision (Figure 4). However, there is no evidence in the literature that such brief interventions produce longer-term effects [43], [44]; but there is evidence in support of telephone counselling in the literature [7] and from the primary trial [17]. If the improvements in the Usual Care group arose in part from the research context, it is unclear whether the same improvements would translate if just the brief intervention components of usual care were applied in a real world context.

Other economic analyses of physical activity and diet behaviour change programs show evidence for cost-effectiveness. Cobiac et al. [45] conducted a modelling study for the Australian setting of six physical activity interventions and found that the use of pedometers and mass media campaigns were likely to be cost-saving and health improving. They also found other programmes like internet-based and general practitioner initiated programmes were cost-effective. Lindgren et al. [46] developed a model of the cost-effectiveness of reducing coronary heart disease events with dietary and exercise advice, for a cohort of 60 year old men in Sweden, showing the intervention was likely to be cost-effective and thus suitable for adoption by health policy makers. Dalziel et al. [13] assessed the cost-effectiveness of a primary care based physical activity counselling intervention in New Zealand. They found that the program cost $NZ 2,053 per QALY gained and recommended that the intervention be adopted broadly. Roux et al. [47] modelled the effectiveness of public health interventions for changing physical activity and estimated subsequent changes to costs and health benefits, measured by QALYs. They found all of the programs to be effective and cost-effective. Tsai et al. [48] used cost and outcome data collected from a trial that recruited severely obese individuals who were randomised to a low carbohydrate or standard weight loss program and concluded the novel diet was not cost-effective. Van Baal et al. [49] assessed the cost-effectiveness of a low-calorie diet, with and without one year of prescribed anti-obesity medication, as compared to no treatment. They recommend adoption of a low-calorie diet alone as the best decision for policy makers. The study reported here adds to small but growing literature on cost-effectiveness of behaviour change interventions.

### Study limitations and strengths

This modelling study has strengths and weaknesses. Predicting the health behaviours of participants for ten years after recruitment has an advantage over trial-based evaluations that only capture events that happen during the period of data collection [50]. Information about the longer-term outcomes that follow a decision to implement the Telephone Counselling program is useful for decision makers and health planners. There are few studies with long-term follow up for health behaviour programs [51], and modelling future outcomes based on observed data is one solution [50]. This advantage is also the source of the major caveat in relation to our findings. Data that describe health behaviour for physical activity and diet for the time between 4 and 12 months were used to predict health-behaviour states for a further nine years, and this may not be accurate. There might be a decline in effectiveness in later years as compared to the changes observed between 4 and 12 months, as the novelty of the intervention wears off. If this is true, then the results are biased in favour of Telephone Counselling. There might, however, be a slower rate of attrition if those who adhered to the relevant health behaviour up to month 12 are more likely to adhere in future time periods. Under this case, the findings on the benefits of Telephone Counselling are conservative. Undertaking longer-term follow-up is the only way to remove this uncertainty; however, this would be an expensive proposition. The modelling assumed the program continued for ten years, and that participants continued to receive scheduled contacts from counsellors and feedback. Thus, the costs of implementing the maintenance part of the intervention for the Telephone Counselling group (month-4 to month-12) were included alongside the costs of Usual Care for 10 years. Because a ten-year trial is unlikely to be funded, a modelling approach to predicting future outcomes is parsimonious.

Risk of death was not reduced in the model when participants adhered to the good health behaviour. There is evidence that improving physical activity and diet reduce risk of premature death [2], [4]. The effect of using an evidence-based reduction in death risk, for those in a state of good health behaviour was tested (results not shown); not surprisingly, the cost-effectiveness of Telephone Counselling increased. The assumption of equal mortality risks for Telephone Counselling and Real Control is therefore conservative, and may underestimate the value of the Telephone Counselling program. Another conservative assumption is that Real Control participants did not deteriorate and cause higher health-care costs, and have worse health outcomes than were observed at baseline. It is likely those with hypertension and diabetes, at the ages observed among the sample, who fail to improve their diet and physical activity, will endure substantial morbidity and health-care costs over a ten-year period. That the sample was recruited from one socio-economically disadvantaged community, and 65% had three or more co-morbidities, suggests that the generalisation of results should proceed with caution. Finally, the model structure was based on health behaviour rather than disease states, because the study participants had a range of co-morbid chronic conditions. This flexible framework might be suitable for other interventions that aim to change health behaviour among a group of participants with heterogeneous health conditions. Choosing Telephone Counselling over existing practice (Real Control) is supported by evidence for efficacy and for cost-effectiveness; with the latter conditional on affordability and opportunity cost [52], [53]. Assumptions about the positive effects being achieved and maintained in broad-reach public health programs can be supported.
